# Health and Human Rights in Eastern Myanmar after the Political Transition: A Population-Based Assessment Using Multistaged Household Cluster Sampling

**DOI:** 10.1371/journal.pone.0121212

**Published:** 2015-05-13

**Authors:** Parveen Kaur Parmar, Charlene C. Barina, Sharon Low, Kyaw Thura Tun, Conrad Otterness, Pue P. Mhote, Saw Nay Htoo, Saw Win Kyaw, Nai Aye Lwin, Cynthia Maung, Naw Merry Moo, Eh Kalu Shwe Oo, Daniel Reh, Nai Chay Mon, Nakul Singh, Ravi Goyal, Adam K. Richards

**Affiliations:** 1 Department of Emergency Medicine, Brigham and Women’s Hospital, Harvard Medical School, Boston, Massachusetts, 02118, United States of America; 2 Community Partners International, Mae Sot, Thailand; 3 University of Washington, Seattle, Washington, United States of America; 4 Burma Medical Association (BMA), Mae Sot, Thailand; 5 Health Information Systems Information Group (HISWG), Mae Sot, Thailand; 6 Back Pack Health Worker Team, Mae Sot, Thailand; 7 Mae Tao Clinic, Mae Sot, Thailand; 8 Karen Department of Health and Welfare, Mae Sot, Thailand; 9 Health Information Systems Information Group (HISWG), Mae Sot, Thailand; 10 Karenni Mobile Health Committee (KnMHC), Mae Hong Son, Thailand; 11 Mon National Health Committee (MNHC), Sangkhlaburi, Thailand; 12 Harvard School of Public Health, Boston, Massachusetts, United States of America; 13 Division of General Internal Medicine and Health Services Research, University of California Los Angeles, Los Angeles, California, United States of America; 14 Community Partners International, Berkeley, California, United States of America; Centre for the AIDS Programme of Research in South Africa (CAPRISA), SOUTH AFRICA

## Abstract

**Background:**

Myanmar transitioned to a nominally civilian parliamentary government in March 2011. Qualitative reports suggest that exposure to violence and displacement has declined while international assistance for health services has increased. An assessment of the impact of these changes on the health and human rights situation has not been published.

**Methods and Findings:**

Five community-based organizations conducted household surveys using two-stage cluster sampling in five states in eastern Myanmar from July 2013-September 2013. Data was collected from 6, 178 households on demographics, mortality, health outcomes, water and sanitation, food security and nutrition, malaria, and human rights violations (HRV). Among children aged 6-59 months screened, the prevalence of global acute malnutrition (representing moderate or severe malnutrition) was 11.3% (8.0 – 14.7). A total of 250 deaths occurred during the year prior to the survey. Infant deaths accounted for 64 of these (IMR 94.2; 95% CI 66.5-133.5) and there were 94 child deaths (U5MR 141.9; 95% CI 94.8-189.0). 10.7% of households (95% CI 7.0-14.5) experienced at least one HRV in the past year, while four percent reported 2 or more HRVs. Household exposure to one or more HRVs was associated with moderate-severe malnutrition among children (14.9 vs. 6.8%; prevalence ratio 2.2, 95% CI 1.2-4.2). Household exposure to HRVs was associated with self-reported fair or poor health status among respondents (PR 1.3; 95% CI 1.1 – 1.5).

**Conclusion:**

This large survey of health and human rights demonstrates that two years after political transition, vulnerable populations of eastern Myanmar are less likely to experience human rights violations compared to previous surveys. However, access to health services remains constrained, and risk of disease and death remains higher than the country as a whole. Efforts to address these poor health indicators should prioritize support for populations that remain outside the scope of most formal government and donor programs.

## Introduction

After holding elections in 2010, Burma/Myanmar (hereafter Myanmar) transitioned to a nominally civilian parliamentary government in March 2011. Since then, Myanmar has seen a dramatic increase in business investment and humanitarian aid [1, 2]. Accurate information evaluating the impact of national efforts to improve health and human rights after the recent political transition is needed.

National health indicators in Myanmar are poor. According to UNICEF, the infant mortality rate (IMR) in Myanmar in 2012 was 41, while neighboring Thailand’s was 11. Similarly, the neonatal mortality rate in Myanmar was 26, as opposed to 8 in Thailand [3]. Official national statistics fail to capture important variation in health status across geographic regions and sub-populations. As a result, these statistics likely underestimate health risks in conflict-affected border regions where state-sponsored data collection and health system responses have been constrained for decades [4].

Since 2002, population-based surveys carried out by community-based organizations (CBOs) with access to these border areas have repeatedly documented rates of disability and death that exceed national estimates. A consortium of CBOs conducted a mortality survey in Shan, Karen, Bago, Karenni, Mon, and Tanintharyi in 2009 that estimated infant (IMR 77) and under-5 mortality rates (U5MR 139) that were substantially higher than national figures reported by UNICEF (IMR 54, U5MR 71) [4]. These surveys demonstrated that in addition to the adverse health effects of rural poverty and poor access to health care, exposure to human rights violations exacerbated health outcomes and shortened lives in Eastern Myanmar prior to the 2010 elections. For example, forced labor has been previously associated with unmet need for contraception [5], child malnutrition and increased infant, child and crude mortality [4, 6]. Food insecurity was associated with maternal anemia and child malnutrition [5, 7], and households experiencing forced displacement were three times more likely to have a child with moderate to severe malnutrition than non-displaced households [4].

Qualitative reports suggest that exposure to human rights violations may have become less common after 2010, as nominally democratic elections and the signing of several ceasefires have ushered in a period of relative decline in active conflict in Eastern Myanmar [8–12]. However, a quantitative survey conducted in 2011 by Physicians for Human rights in Karen State found that one quarter of households experienced at least one HRV in the previous year and that exposure to HRVs continued to be associated with poor health outcomes. Odds of reporting household hunger were nearly ten times higher (OR 9.9) for families who had experienced assault the preceding year, and 4.6 times higher among families who reported that food had been stolen or destroyed in the past 12 months [13].

International assistance for health services has increased since 2010 [1, 2]. However, a comprehensive assessment of the impact of these changes on the health and human rights situation has not been published. This manuscript presents the results of a multi-state household survey designed to describe the health situation in five states in Eastern Myanmar three years after the political transition, and to quantify contemporary HRV exposures and their possible impact on health. Katchin State and Shan State, two regions that have faced more active conflict over the past several years, were not included in this study.

## Methods

### Design

From July 2013-September 2013, 80 surveyors conducted retrospective household surveys in five states and regions, including securely and physically accessible areas of Bago, Karen, Karenni, Tanintharyi, and Mon. Similar to a 2009 survey [4], the primary objective of the study was to estimate morbidity and mortality in each service areas of five CBOs that deliver health services to internally displaced persons (IDPs) and other remote populations in eastern Myanmar. Additional outcomes of interest included demographics, migration, mortality, self-reported health status, reproductive health, child health, water and sanitation, food access and nutrition, malaria, human rights violations, and access to health services.

The sampling frame of 456,786 people (87,841 households) was constructed using village-level population lists provided by CBOs that had been updated within the previous year. Geographic boundaries were drawn based on service (or catchment) areas for each CBO. The stratified, two-stage household sampling protocol was designed to facilitate estimation of under-five mortality rates in each CBO service area (stratum). In the first stage, clusters were selected using population proportional to size (PPS); in the second stage, proximity sampling was used to select 30 households for each cluster. A household was defined as a group of people who live under the same roof for two or more months and share meals.

### Implementation

Surveyors chosen from CBO staff participated in a two-week training conducted in the local language in one of three locations. The training covered sampling, interviewing techniques, informed consent protocols, use of global positioning (GPS) units, handling adverse events, mid-upper arm (MUAC) measurement, and rapid diagnostic testing for *Plasmodium falciparum* (Pf) malaria. Instruction was complemented with mock interviews and observed field piloting of the survey tool.

The survey instrument was originally written in English, then translated to Burmese, Mon, and Sgaw Karen and then each of these languages was back-translated into English. The survey asked respondents to enumerate the age, sex, and in/out-migration of all household members and give the age and perceived cause of death of all who had died in the household in the past year with the exception of miscarriages, abortions, and stillbirths. Acute malnutrition was assessed by measuring mid-upper arm circumference (MUAC) of women of reproductive age (WRA; 15–49 years) and children 6–59 months of age. All members of the first, 15^th^, and 30^th^ household were tested with Paracheck (Goa, India) rapid diagnostic test for infection with Pf malaria. In clusters with less than 30 households, another household was chosen at random to replace the 30^th^ household.

Interviewers requested that the head of household (male or female) respond to the first 78 survey questions. If the head of household was unavailable, respondents were selected in the following descending order of priority: WRA with the youngest child under five in the household, WRA currently pregnant, then oldest WRA. In order to increase the number and fidelity of data collected about pregnancy history, interviewers asked an additional 19 reproductive health questions of all WRAs in the household who either had a child under five or were pregnant at the time of the survey.

Recall periods for most questions were for the past 12 months, with the following exceptions: diarrhea and ORS usage in children under 5 (2 weeks), vitamin A and deworming for children under 5 (6 months), reproductive health histories (last pregnancy), landmine injuries (12 months and 15 years), and clean drinking water and mosquito net usage (24 hours).

### Analysis

Crude mortality and age-specific death rates (ASDR) were calculated using a ratio of deaths to mid-year population. U5MR and IMR were calculated as a ratio of deaths per thousand live births using standard approaches. Morbidity outcomes were analyzed with prevalence calculations. For counts of deaths within households, rate ratios were estimated using Poisson regression with an offset for the number of household members at-risk for outcome events within relevant age groups.

MUAC data for children ages 6 to 59 months were analyzed using two complementary approaches. First, commonly used MUAC cutoffs were used to categorize children with severe (<11.5 cm), moderate (11.5 to <12.5cm) and mild (12.5 to <13.5cm) acute malnutrition. Second, in order to facilitate comparison with previous surveys in Eastern Myanmar we used World Health Organization (WHO) Child Growth Standards to calculate MUAC-for-age z-scores as recommended by WHO [14, 15]. Children with MUAC measurements less than -1, -2 or -3 standard deviations below their age- and sex-specific mean (Z-score) were categorized as mildly, moderately and severely malnourished, respectively. In the analysis of women of reproductive age, MUACs less than 22.5cm were considered malnourished.

All analyses were conducted using post-stratification weights; post-stratification weights were equal to the inverse sampling probability of being in a selected household. Data analysis was conducted using R [16], employing the survey package [17] to account for the design effect due to clustering at the village level in standard error calculations.

In bivariate analyses, households that did not experience the specific abuse under scrutiny were included to estimate the risk of adverse health outcomes. In order to assess associations between health outcomes and exposure to HRVs, generalized linear models with a log link function that account for design effect were used to compare households with no HRV exposure to households with one or more.

### Sample Size

The sample size was based on a balance between operational feasibility under security and resource constraints and the goal for continued monitoring of the U5MR with reasonable precision within each CBO service area (stratum). The sample size of 1,350 households in each stratum allows for precision of U5MR for each CBO service area to within 50/1000 live births, assuming an U5MR = 138/1000, a crude birth rate = 38/1000, mean household size of 5.2, survey completion rate of 90%, design effect of 2.0 [4–6] and an alpha of 0.1 (90% CI). The overall sample size (n = 6,750 households in 225 clusters in 5 strata) allows for a precision of overall U5MR to within +/- 30/1000, assuming a slightly larger design effect (2.5) to account for between-stratum variance, and an alpha of 0.05 (95% CI).

### Ethical approval

A verbal informed consent process was undertaken with each head of household. Due to the sensitive nature of some elements of the survey, only verbal consent was obtained to protect the respondents. When cases of malaria or malnutrition were uncovered, or when respondents expressed distress resulting from questions asked, surveyors referred affected individuals to local community leaders to seek necessary care. All participants provided informed verbal consent, which was documented on returned paper surveys by surveyors. Heads of household over the age of 15 were interviewed. As heads of household in Eastern Burma are occasionally between the ages of 15–18, and live away from their parents, no additional consent was sought from next of kin. The Institutional Review Boards at Partners HealthCare and the University of California Los Angeles provided ethical review and approved the study protocol, including the verbal consent process.

## Results

Out of 225 clusters planned (6,750 households), security concerns led to the replacement of 10 clusters, and prevented surveys from being conducted in 6 clusters. The final sample analyzed included 219 clusters (29 clusters had fewer than 30 households in the village), with 30,323 people enumerated in 6,178 households. The overall response rate was 91.5% [Table 1].

**Table 1:** Surveys Returned.

Target population	456,786
Number of clusters sampled	225
Number of clusters reached	219
Total households sampled	6,750
Total households reached	6, 220
Total consenting households	6, 178
Total population in consenting households	30,323
Overall response rate (consented / sampled households)	91.5%

### Demographics

Roughly fifteen percent of the sampled population was under 5 (14.6%; 95% CI 13.8–15.4). Mean household size was 4.9, and the overall male to female ratio was 0.97. The male to female ratio was lower among ages 15–25 (0.93), consistent with previous surveys in eastern Myanmar and other conflict-affected regions where young men are lost to war or migration [4]. A majority of those sampled identify as ethnically Karen (66.9%; 95% CI 60.6–73.3) and speak Sgaw Karen (60.2%; 95%CI 53.3–67.2), though a substantial proportion also speak Burmese (41.4%; 95% CI 36.1–46.7). A majority of the sample identified themselves as Buddhist (58.5%; 95% CI 51.6–65.5), though a substantial minority identified as Christian (35.0%; 95% CI 28.3–41.7). Level of education among respondents was very low in this region, as 42.3% of men (95% CI 38.3–46.2) and 35.3% of women (95% CI 30.4–40.2) reported having had no formal education. Among children under five, 53.6% (95% CI 47.9–59.4) had no official birth record. By virtue of a recent initiative by local CBOs, 32.3% (95% CI 26.0–38.5) reported holding an “ethnic” birth record, which refers to birth records issued by an ethnic CBO [Table 2].

**Table 2:** Demographics of enumerated individuals (N = 30,323).

Characteristic	Proportion (95% CI)
Male	49.7% (48.9–50.4)
Female	50.3% (49.6–51.1)
Male Respondents	46.6% (42.3–50.9)
Female Respondents	53.4% (49.1–57.6)
Mean household size	4.9 (4.8–5.1)
Population <5 years old (%)	14.6% (13.8–15.4)
Population <15 years old (%)	40.2% (38.8–41.5)
Population >65 years old (%)	3.6% (3.1–4.1)
Male to female ratio (all ages)	0.97
Male to female ratio (15–25 years)	0.93
Language*	
Pwo Karen	15.1% (10.1–20.1)
Sgaw Karen	60.2% (53.3–67.2)
Burmese	41.4% (36.1–46.7)
Shan	5.4% (1.7–9.1)
Karenni	7.7% (6.3–9.2)
Mon	10.4% (6.8–14.1)
Other	8.8% (4.3–13.3)
Ethnicity	
Karen	66.9% (60.6–73.3)
Karenni	10.5% (7.0–14.0)
Shan	3.4% (0.2–6.6)
Mon	10.6% (7.0–14.2)
Burmese	0.8% (0.4–1.2)
Other	7.5% (3.1–12.0)
Religious affiliation	
Christian	35.0% (28.3–41.7)
Buddhist	58.5% (51.6–65.5)
Muslim	0.1% (<0.1–0.3)
Animist	5.7% (3.4–8.1)
None	<0.1% (<0.1-.1)
Educational attainment of respondents	
Male	
None	42.3% (38.3–46.2)
1 to 5 standard	37.1% (33.7–40.6)
6 to 10 standard	13.2% (11.3–15.1)
Above 10 standard	1.5% (1.0–2.0)
Other education (Short course/ Monastery)	5.1% (3.8–6.4)
Don't know	0.2% (0.1–0.3)
Refused	0.4% (0.03–0.8)
Female	
None	35.3% (30.4–40.2)
1 to 5 standard	39.6% (35.1–44.0)
6 to 10 standard	14.9% (12.5–17.3)
Above 10 standard	1.4% (0.7–2.1)
Other education (Short course/ Monastery)	8.2% (5.7–10.7)
Don't know	0.1% (<0.1–0.3)
Refused	0.3% (<0.1–0.8)
Access for Official Documents Under Age 5	
No birth record	53.6% (47.9–59.4)
Ethnic birth record	32.3% (26.0–38.5)
Government birth record	7.9% (5.8–9.9)
Other country’s birth record	1.7% (1.0–2.4)

* respondents may have selected more than one language for this question

### Morbidity

Self-reported fair or poor health is associated with mortality across a range of cultural and economic settings [18–21]. A majority of respondents (56%) reported they were in fair or poor health; 9.4% stated their health was poor. Each respondent was asked the Patient Health Questionnaire 2 (PHQ-2), a 2-item depression screen validated to identify individuals at risk for depression across a range of cultural, linguistic and country settings [22–25]. In this sample, five percent of respondents (95% CI 3.7–6.4) screened positive for depression, based on a commonly used threshold of four or higher on a scale of zero to six. Every individual in the 1^st^, 15^th^, and 30^th^ household in each cluster was offered a test for Pf malaria, and 2269 agreed to be tested. Of these, 2.3% (95% CI 1.2–3.4) tested positive.

Among 2768 children between 6–59 months of age screened for malnutrition (68.2% of all children within that age range), the prevalence of global acute malnutrition was 11.3% (95% CI 8.0–14.7) using MUAC cutoffs and 16.8% (95% CI 13.0–20.6) using WHO MUAC-for-age z-scores. Nearly 20% of children had had diarrhea in the 2 weeks prior to the survey (19.8%; 95% CI 16.7–22.9), and 39.1% of these children with diarrhea received oral rehydration solution (ORS; 95% CI 31.7–46.4). Parents reported 37.5% (95% CI 32.1–42.8) of their children took Vitamin A pills and 50.1% (95% CI 45.0–55.2) took deworming medication in the 6 months prior to the survey [Table 3].

**Table 3:** Prevalence of morbidities.

Outcomes	Overall (95% CI)
Self-reported health status (n = 6132)	
Good	44.0% (39.4–48.5)
Fair	45.9% (41.7–50.2)
Poor	9.4% (7.4–11.3)
Don't know	0.1% (<0.01–0.2)
Refused	0.6% (<0.01–1.1)
Fair or Poor	55.5% (51.2–59.7)
Plasmodium falciparum positive (n = 2269)	2.3% (1.2–3.4)
PHQ-2 Depression Screen (n = 6082)	
0 to 3	95.0% (93.6–96.3)
4 to 6	5.0% (3.7–6.4)
CHILD HEALTH (<5) (n = 4059)	
Acute child malnutrition (among children 6–59 months, n = 2768 assessed)	
MUAC cutoffs	
Mild (12.5 to <13.5cm)	14.7 (12.3–17.2)
Moderate (11.5 to <12.5cm)	6.2 (4.5–8.0)
Severe (<11.5cm)	5.1 (2.8–7.4)
Global acute malnutrition (GAM = moderate + severe malnutrition)	11.3 (8.0–14.7)
MUAC-for-Age (WHO Z-scores)	
Mild	24.4% (21.4–27.5)
Moderate	10.3% (8.2–12.3)
Severe	6.5% (3.8–9.3)
GAM	16.8% (13.0–20.6)
Child diarrhea in previous 2 weeks	19.8% (16.7–22.9)
Child with Diarrhea Given ORS	39.1% (31.7–46.4)
Provision of Vitamin A pills *	n = 131137.5% (32.1–42.8)
Provision of deworming pills **	n = 163250.1 (45.0–55.2)

*Among children older than 6 months and less than 5 years

**Among children older than 1 year and less than 5 years

### Reproductive Health

The crude birth rate in this population was 28.9 per thousand people per year (95% CI 25.9–32.4); and the general fertility rate was 139.5 per thousand women of reproductive age (95% CI 123.4–155.6). Eleven percent of reproductive aged women (11.3; 95% CI 9.1–13.5) were malnourished, based on a MUAC measurement of 22.5cm or less. Women of reproductive age (15–49 years) who were either pregnant or had children under five years of age were asked several questions on reproductive health. Of women who were asked, those who reported using contraceptives overwhelmingly used hormonal methods, including depot injections (65.8%; 95% CI 60.2–71.3) and oral contraceptives (28.8%; 95% CI 23.8–33.7). Unmet need for contraception was defined as the proportion of women not currently using contraceptive methods among those who stated they were not planning to have more children, or who were not currently pregnant and did not explicitly state that they did not need contraception. By this definition, “unmet need” for contraceptives among women with young children was 54.1% (95% CI 48.9–59.2) [Table 4].

**Table 4:** Reproductive Health *.

Indicator	Overall (95% CI)
Crude birth rate	28.9 (25.9–32.4)
General Fertility Rate (among 8334 women of reproductive age)	139.5 (123.4–155.6)
Malnutrition among Women of Reproductive Age (15–49 years old n = 5142 with valid MUAC measurement)	11.3% (9.1–13.5)
Respondents who do not plan on having more children	40.7% (37.–44.5)
Mean number of lifetime pregnancies	3.9 (3.7–4.1)
Mean age at first pregnancy	21.5 (21.2–21.8)
Unmet Need for contraception** (N = 1144)	54.1% (48.9–59.2)
Contraception use	26.7% (23.1–30.3)
Of those using contraception (N = 1080)	
Oral pills	28.8% (23.8–33.7)
Depo injection	65.8% (60.2–71.3)
IUD	1.0% (0.3–1.7)
Norplant	0.1% (<0.01–0.3)
Male condom	1.2% (<0.01–2.5)
Female condom	0.3% (<0.01–0.7)
Sterilization	4.3% (2.4–6.2)
Calendar method/withdrawal/abstinence	1.0% (<0.01–2.4)
Exclusive breastfeeding	0 (0–0)
Traditional medicine/method	0.5% (<0.01–1.1)
Other	0.1% (<0.01–0.3)
Pregnancy care since political transition (past two year, n = 2062)	
Four or more ANC visits during last pregnancy	16.4% (12.4–20.4)
Who provided antenatal care during last pregnancy?	
Doctors/Nurses	10.2% (6.8–13.6)
Health Assistants/MW/AMW	12.5% (7.7–17.3)
Ethnic health workers/medic	36.4% (29.6–43.1)
TBA	50.7% (42.4–58.9)
Others	7.4% (3.2–11.5)
Use of Iron/Folate Supplementation During Pregnancy (any)	60.9% (55.8–66.0)
Use of Iron/Folate Supplementation During Pregnancy (at least 90 days)	9.9% (7.5–12.3)
Use of deworming pill during pregnancy (any)	34.0% (28.2–39.9)
Birth Attendants	
Doctors/Nurses	5.2% (3.2–7.2)
Health Assistants/MW/AMW	5.4% (3.2–7.6)
Ethnic health workers/medic	11.4% (7.7–15.0)
TBA	73.0% (67.5–78.5)
Others	14.4% (9.4–19.5)
Number of Post-natal care (PNC) visits	
No PNC visit	52.0% (45.1–59.0)
1–3 visit(s)	36.2% (29.7–42.7)
4 or more visits	7.5% (3.6–11.4)
DK/NA/Refused/Missing	4.3% (2.9–5.6)
Breastfeeding duration	
Did not breastfeed	0.2% (<0.01–0.5)
3 months and less	2.5% (1.4–3.7)
4–6 months	1.2% (<0.1–2.9)
7–9 months	0.6% (0.2–1.1)
More than 9 months	17.0% (11.4–22.6)
Still breastfeeding	74.8% (68.7–80.9)
DK/NA/Refused/Missing	3.6% (2.1–5.1)
Exclusive breastfeeding: age when introduced liquid/other supplementary foods	
0–6 months	36.4% (30.9–41.9)
After 6 months	37.3% (31.4–43.1)
Still exclusively breastfeeding	21.9% (17.6–26.1)
DK/NA/Refused/Missing	4.5% (2.9–5.6)

* Responses available for women currently pregnant or with children under age 5.

**Unmet need = (those who reported not using contraception) / (women who do not plan for more children AND who are not currently pregnant AND who did not say contraception was "not needed").

Women of reproductive age who were pregnant or who had children under five years of age were asked about pregnancy history. Surveyed women reported a mean of 3.9 pregnancies (95% CI 3.7–4.1) during their lifetime with a mean age at first pregnancy of 21.5 years (95% CI 21.2–21.8). Among women pregnant since political transition (in the two years prior to the interview, n = 2,062), 16.4% (95% CI 12.4–20.4) met the international standard of four or more antenatal visits during their last pregnancy [26]. Access to trained health professionals for antenatal and intra-partum care was limited. Only a small proportion of women reported access to doctors or nurses during their last pregnancy (10.2%; 95% CI 6.8–13.6). Women were most likely to report having received antenatal care from a traditional birth attendant (TBA, 50.7%; 95% CI 42.4–58.9) or ethnic CBO health worker (36.4%; 95% CI 29.6–43.1). The majority of women (73.0%; 95% CI 67.5–78.5) reported that TBAs had assisted at their most recent birth whereas 5.2% (95% CI 3.2–7.2) of women reported having a doctor or nurse present during the birth of their last child. Fifty-two percent of women reported no post-natal care (95% CI 45.1–59.0) [Table 4]. Adequate iron/folate supplementation during pregnancy was rare (9.9%; 95% CI 7.5–12.3 reported at least 90 days) [27], and one-third of women (34.0%; 95% CI 28.2–39.9) reported taking deworming medication. Most women had breastfed their infants for at least six months or were breastfeeding at the time of the survey, and the majority reported exclusive breastfeeding for at least six months. Approximately one-third (36.4%; 95% CI 30.9–41.9) introduced supplementary foods before six months of age.

### Mortality

A total of 991 live births and 250 deaths occurred during the year prior to the survey. Infant deaths accounted for 64 of these (IMR 94.2; 95% CI 66.5–133.5) and there were 94 child deaths (U5MR 141.9; 95% CI 94.8–189.0). The crude mortality rate (CMR) was 9.2 (95% CI 7.2–11.8) [Table 5]. Respondents attributed 17.7% of deaths to malaria (95% CI 9.3–26.1), 14.0% to acute respiratory tract infection (ARI, 95% CI 8.3–19.7) and 10.4% (95% CI 4.0–16.8) to diarrhea. There were 2 deaths attributed to gunshots, 2 to landmines, and one related to pregnancy in this sample. Neonatal deaths accounted for the greatest proportion of deaths under 5 (36.5%; 95% CI 21.9–51.1), with diarrhea, ARI, and malaria accounting for a majority of remaining deaths [Table 6].

**Table 5:** Mid-year population estimates, vital events, mortality rate estimates.

	Overall (95% CI)
Vital events	
Crude birth rate	28.9 (25.9–32.4)
Live births	991
Infant deaths	64
Child deaths	94
Total deaths	250
Mortality rates/ratios	
Infant (IMR)	94.2 (66.5–133.5)
Child (U5MR)	141.9 (94.8–189.0)
Child (ASDR-5)	26.4 (19.2–36.6)
Overall (CMR)	9.2 (7.2–11.8)

Weighted parameter estimates and their standard errors (and confidence intervals) account for the complex survey design.

**Table 6:** Reported Causes of Death.

	Count	Proportion	Overall (95% CI)
All Ages (n = 250)			
Malaria	38	17.7%	(9.3–26.1)
ARI	35	14.0%	(8.3–19.7)
Diarrhea	27	10.4%	(4.0–16.8)
Neonatal	36	15.6%	(8.4–22.9)
Pregnancy-related	1	0.7%	(<0.1–2.0)
Gunshot	2	1.1%	(<0.1–2.6)
Landmine	2	0.9%	(<0.1–2.4)
Don't know	28	10.1%	(5.7–14.6)
Other	81	29.4%	(20.1–38.7)
Under 5 (n = 94)			
Malaria	11	14.8%	(0.1–29.5)
Diarrhea	16	15.6%	(5.6–25.6)
ARI	13	11.8%	(5.5–18.2)
Neonatal	36	36.5%	(21.9–51.1)
Other	11	12.8%	(3.6–22.0)
Don't know	7	8.5%	(0.5–16.5)

### Human Rights Violations

All respondents were asked to report on exposure to human rights violations (HRVs) within the past year using a module previously developed for use in the region [4, 6]. Approximately one in nine households (10.7%; 95% CI 7.0–14.5) experienced at least one HRV in the past year and four percent reported two or more HRVs [Table 7]. The most common HRVs reported were destruction and seizure of food, livestock, or crops (7.7%; 95% CI 4.1–11.4) and forced labor (3.5%; 95% CI 1.4–5.5).

**Table 7:** Prevalence of various human rights violations.

Violation	Overall (95% CI)	Number of individuals reporting this event
Forced labor	3.5% (1.4–5.5)	246
Destruction and seizure of food, livestock, or crops	7.7% (4.1–11.4)	443
Confiscation of land	0.8% (0.2–1.4)	81
Physical injuries (gunshot wounds, landmine injuries, beatings, stabbings)	0.2% (0.03–0.29)	7
Detained or tied up	<0.1%	2
Landmine injury in last 12 months	<0.1%	5
Landmine injury in past 15 years	5.3% (3.4–7.3)	271
Number of human rights violations experienced by household		
0	89.3% (85.5–93.0)	5501
1	6.7% (4.0–9.4)	407
2	1.8% (1.0–2.7)	111
3	1.9% (0.2–3.7)	139
4	0.3% (0.1–0.5)	19
5 or more	<0.1%	1
Any human rights violation	10.7% (7.0–14.5)	677

Weighted prevalence estimates and their standard errors (and confidence intervals) account for the complex survey design.

### Health and Human Rights

Associations between morbidity and mortality outcomes and household exposures to human rights violations were assessed using prevalence and rate ratios respectively. As has been found in previous studies in eastern Myanmar [4, 6], household exposure to one or more HRVs was associated with global acute malnutrition among children using either MUAC cutoffs (14.9 vs. 6.8%, prevalence ratio 2.2; 95% CI (1.2–4.2)) or MUAC-for-age (17.7 vs. 10.1%, prevalence ratio 1.7; 95% CI (1.1–2.8)) [Table 8]. Household exposure to HRVs also was associated with self-reported fair or poor health status among respondents (PR 1.3; 95% CI 1.1–1.5). However, HRVs were not associated with a more strict definition of poor health (limited to individuals reporting their health as poor, PR 0.9). Point estimates suggest that exposure to HRVs may also be associated with increased risk of child death (ASDR-5 36.9 vs. 25.0 per 1,000, PR 1.5); however, the absolute number of deaths among children in exposed households was small (n = 17) and results did not reach statistical significance (95% CI 0.8–2.9). Household exposure to HRVs was not associated with depressive symptoms, access to food, or child diarrhea.

**Table 8:** Morbidity and Mortality Associated with Household Exposure to Human Rights Violations.

*Outcome*	*Deaths*	*Respondents*	Deaths/population * 1000	*Rate Ratio*
*ASDR-5*	*unit of analysis—children under 5*			
*No violations reported*	*77*	*3707*	*25*.*3*	
*≥1 violation*	*17*	*446*	*37*.*8*	*1*.*5 (0*.*8–2*.*9)*
*CMR*	*unit of analysis—total population*			
*No violations reported*	*212*	*26987*	*9*.*0*	
*≥1 violation*	*38*	*3586*	*10*.*7*	*1*.*2 (0*.*7–2*.*0)*
*Under-5 Diarrhea*	*unit of analysis—children under 5*			
*No violations reported*	*656*	*3630*	*18*.*6*	
*≥1 violation*	*98*	*429*	*21*.*5*	*1*.*2 (0*.*8–1*.*8)*
*Moderate-Severe Malnutrition*: *MUAC <12*.*5cm*	*unit of analysis—children under 5*			
*No violations reported*	*218*	*3630*	*6*.*8*	
*≥1 violation*	*61*	*429*	*14*.*9*	*2*.*2 (1*.*2–4*.*2)*
*Moderate-Severe Malnutrition*: *MUAC-for-Age (WHO z-score <2)*				
*No violations reported*	*352*	*3708*	*10*.*1*	
*≥1 violation*	*81*	*446*	*17*.*7*	*1*.*7 (1*.*1–2*.*8)*
	*Cases*	*Respondents*	*%*	*Prevalence Ratio*
*Depression score >3*				
*No violations reported*	*298*	*5504*	*5*.*1*	
*≥1 violation*	*33*	*677*	*4*.*7*	*0*.*9 (0*.*5–1*.*6)*
	*Cases*	*Respondents*	*%*	*Prevalence Ratio*
*Self-reported general health (fair/poor)*				
*No violations reported*	2606	5504	54.2	
*≥1 violation*	406	677	69.1	1.3 (1.1–1.5)
*Self-reported general health (poor)*				
*No violations reported*	*495*	*5504*	9.6	
*≥1 violation*	*54*	*677*	8.4	*0*.*9 (0*.*6–1*.*4)*

Prevalence ratios were estimated using weighted generalized linear models that accounted for the complex survey design. The unit of analysis was the household or individual respondent unless otherwise specified. The mortality rate ratios accounts for the number of household members at risk by using Poisson regression with an offset.

## Discussion

This large cross-sectional survey demonstrates that populations in eastern Myanmar continue to experience a high risk of death, disease and human rights violations two years after political transition. The survey represents a coordinated research effort of multiple CBOs to document the health, mortality, and human rights situation. Findings are consistent with qualitative reports of a reduction in human rights violations (HRVs) in Eastern Myanmar since political transition; however, these modest gains have not been accompanied by concomitant improvements in health services and outcomes, particularly among children and women.

### Mortality

Mortality rates among infants (IMR 94.2; 95% CI 66.5–133.5) and children (U5MR 141.9; 95% CI 94.8–189.0) were similar to those estimated in 2009 (IMR 77; U5MR 139)[4] and remained substantially higher than official statistics reported for the country as a whole in 2012 (IMR 41, U5MR 52)[28]. Although variance estimates for national mortality figures are unavailable, both the point estimates and lower confidence limits for IMR and U5MR in the present survey were substantially higher than national point estimates. This makes it unlikely that infant and child mortality rates in eastern Myanmar were similar to countrywide estimates. It is not possible to determine the extent to which the estimated mortality differences reflect a true elevation in risk of death in Eastern Myanmar or if remote border populations were under-represented in the surveys used to derive national figures.

Although the crude mortality rate estimated for the overall sample population (9.2 per 1,000; 95% CI 7.2–11.8) is similar to the officially reported rate for the country as a whole (8.2) [29], direct comparison should be made with caution given that rates are not age-standardized, and the study population has a substantially younger age distribution than that of Myanmar as a whole. Children under 5 comprised 14.6% of individuals in the present study compared to 8.4% of the Myanmar national population. Adults (age 15–64) and elderly (65 and over) make up less than two thirds (56.2 + 3.6 = 59.8%) of the current study population, compared to three-fourths of people in Myanmar overall (70 + 5 = 75%) [30, 31]. Consistent with previous surveys, the majority of deaths in Eastern Myanmar are reported to be due to preventable causes [6, 32].

### Child malnutrition and morbidity

Children in eastern Myanmar face a critical health situation that appears not to have improved with the recent political transition. The prevalence of global acute malnutrition (GAM) in Eastern Myanmar is higher than GAM reported for more stable areas of Myanmar and for the country as a whole (7.9%)[28]. Using MUAC cutoffs, the present survey estimates GAM as 11.3% (95% CI 8.0–14.7), which falls within the “severe” range of GAM as defined by the World Health Organization (10–14%). This more than three times higher than recent estimates from Myanmar’s Dry Zone using the same approach (3.1%) [33]. Using the MUAC-for-age approach suggested by WHO, the present estimate is similar to that calculated using comparable methods in a survey in Eastern Myanmar in 2009 (14.8%; 95% CI 11.5–18.1) [4] and may now exceed the 15% threshold considered a “critical” level of malnutrition by WHO [34]. The lower bound of the 95% confidence intervals using either approach in the present survey (8.0% and 11.5%) excludes the GAM point estimate for the country as a whole (7.9%); and levels of malnutrition appear to more closely resemble those of Rohingya living in internally displaced person (IDP) camps in Rakhine state where GAM was found to be 14.4% [35]. However, alternative methods to assess acute malnutrition in populations such as weight-for-height Z score (WHZ), MUAC cutoffs or MUAC-for-age, are not equivalent and direct comparison should be made with caution [36, 37]. For example, a survey conducted in the Myanmar Dry Zone in 2013 produced divergent estimates of GAM when MUAC cutoffs (GAM 3.1%) and WHZ (12.3%) were used. It is notable that the prevalence of severe acute malnutrition (SAM) in the dry-zone survey was very low, irrespective of the method used (0.2% by MUAC; 0.5% by WHZ). In contrast, both methods employed in the present study in Eastern Myanmar (and) produced estimates of SAM that are nearly an order of magnitude higher (5.1% using MUAC cutoffs and 6.5%, MUAC-for-age), and comparable to levels of SAM documented among Rohingya IDPs (4.5% using MUAC cutoffs).

The Karen Department of Health and Welfare previously piloted a successful population-based program to screen and provide therapeutic and supplemental food for acutely malnourished children in eastern Myanmar [38], but a substantial increase in resources is required in order to scale-up programs to prevent, identify, and treat hunger throughout the region.

The percentage of children with diarrhea in the previous two weeks (19.8%) was higher than countrywide estimates (7%); however, some of this difference may be attributable to the timing of the survey, which was conducted during the peak of the rainy season (June-July). A survey conducted in a similar population in eastern Burma during the dry season (October 2008 to January 2009) found a lower percentage of children with recent diarrheal illness (10%)[4].

Access to essential medications among children was low, with a minority reporting child receipt of Vitamin A (36.8%) or deworming medicine (eg. Albendazole, 45.7%). Only 39.1% of children with diarrhea received ORS. This proportion is lower than the official estimate among rural children in Myanmar (56%)[28].

### Health of women

Reproductive health indicators suggested poor access to antenatal care, with 16.9% of women reporting four or more antenatal care visits [26] during their last pregnancy. This is substantially lower than the officially reported percentage (73.4%)[28]. A small fraction of women saw doctors or nurses during their last pregnancy or delivery [Table 4], while the vast majority reported receiving care from ethnic minority CBO health workers or TBAs [Table 4]. Eleven percent of women reported having taken adequate amounts of iron supplementation during their last pregnancy, and over half of women reported an unmet need for contraceptives (54.1%). The prevalence of under-nutrition among women of reproductive age was similar to that estimated in 2009 (16.7%)[4]. These findings suggest that access to government sanctioned reproductive health services continues to be extremely poor in eastern Myanmar. Ethnic CBO health workers and TBAs play an important role in addressing large delivery gaps, but substantial resources and effort is required to expand successful models of community based maternal care [5, 7, 39, 40], and to strengthen linkages with institutions capable of providing higher level care in case of complications.

### Human rights

The improved human rights situation is suggested by a substantial decrease in the prevalence of human rights violations. In the present survey, 10.7% of households reported experiencing at least one human rights violation in the previous 12 months. In contrast, population-based surveys conducted in Eastern Burma in 2009 and in Karen State in 2011 found that one third of households [4, 13] had experienced one or more HRVs in the previous 12 months. The most dramatic improvement in the human rights situation since the 2010 elections appears to be related to a decrease in arbitrary detention and overt acts of physical violence such as gunshot wounds, beatings, and torture. Whereas 103 households interviewed for the 2009 survey reported being detained by authorities (2.5%; 95% CI 1.0, 4.1) and 217 households (5.0%; 95% CI 3.8–6.2) reported physical injuries due to violent HRVs [4], very few households reported detention ([n = 2] households, <0.1%) or exposure to violence ([n = 7] households, 0.2%; 95% CI 0.03–0.3) in the present survey conducted in 2013 (Table 8). Exposure to forced labor also was less frequently reported in 2013 (3.5% of households reported forced labor during the prior year; 95% CI 1.4–5.5) than in 2009 (13.3%; 95% CI 10.1–16.5) and fewer households reported destruction and seizure of food, livestock, or crops (7.7%; 95% CI 4.1–11.4 vs. 16.1%; 95% CI 12.0, 20.1 in 2009) [4]. Less than 1% of respondents reported confiscation of land (0.8%; 95% CI 0.2–1.4). These findings are encouraging and consistent with qualitative reports suggesting that eastern Myanmar has become less violent since the signing of several tentative ceasefires [9]. However, it is important to note that areas subject to high levels of insecurity due to ongoing conflict were excluded from this survey. These areas may have a higher prevalence of human rights violations.

### Human Rights and Health

Despite promising signs of progress, widespread exposures to HRVs remain. Though there was a relative decline in incidence, HRVs continued to be associated with adverse health outcomes including child malnutrition (PR 2.2 using MUAC cutoffs; 1.7 using MUAC-for-Age) and self-reported fair or poor health status (PR 1.3). The present survey was not designed to predict health outcomes under an unobserved counterfactual scenario under which eastern Myanmar was free of HRVs. However, the fact that moderate and severe malnutrition was documented in 6.8% (using MUAC cutoffs; 10.1% using MUAC-for-age) of children living in households that did not experience an HRV during the previous year suggests that the severe (or critical) malnutrition threshold of 10% (or 15%) is attributable to the exceptional rate of malnutrition among the 10.7% of households that were exposed to HRVs (14.9% using MUAC cutoffs; 17.7% using MUAC-for-age).

The present survey did not replicate findings from previous surveys that had documented associations between HRVs and several other health outcomes, including depression [41, 42] and child diarrhea [4]. Among possible explanations for the absence of an association with the PHQ-2 depression screen is the low frequency of displacement or cumulative exposure to multiple violent or traumatic events that have demonstrated the strongest associations with mental health outcomes. As noted above, in contrast to the previous study that found an association between HRVs and child diarrhea, the present survey was conducted during the rainy season, when elevated incidence of diarrhea overall may obscure a marginal impact of HRVs. Associations between HRVs and malaria were not estimated due to the very low prevalence of malaria (2.3%) that approximated the false positive rate of the rapid diagnostic test (complement of test specificity of less than 98%).

### Health Access and Birth registration

This survey was not designed to elucidate etiologic mechanisms or describe comprehensive causal pathways to explain the increased risk of death and illness in Eastern Burma, even among households that were not exposed to HRVs. However, several indicators highlight the vulnerable position of the majority of households, and suggest that many communities remain outside the reach of most official government and development programs. First, this study found poor access to government health services, highlighted by the low proportion of women with access to antenatal care and health attendants during their last childbirth [Table 4].

The second marker of marginalized status of sampled households is the low rate of official birth registration. In contrast to the officially reported rate of birth registration in Myanmar (72% [3]) only 7.9% of children in these populations in Eastern Myanmar have an official government birth record. Unless and until the remainder is registered, up to 92% of children are *de facto* stateless persons. Statelessness results in decreased access to education, health care, access to voting rights [43]. Ethnic minority organizations, including the Back Pack Health Worker Team and Karen Department of Health and Welfare, have issued birth certificates, and this survey suggests that approximately one third (32.3%) of children already have a CBO birth record. If formally recognized, these birth records would allow children have the benefits of an official birth record until a durable solution to unregistered births is identified.

### Limitations

While this study’s cross-sectional design precludes causal inference, feasible causal pathways between HRVs and health exist and have been described elsewhere [6]. All causes of death reported by surviving household members are subject to misclassification, and were not subjected to verbal autopsy or physician certification. In order to minimize information bias, surveys were administered in four local languages. Surveyors were trained to use locally familiar dates as time anchors to minimize recall bias, though recall bias remains possible, particularly for mortality outcomes.

As the current survey’s HRV module did not include questions concerning the duration or frequency of exposures, perpetrator, or timing of events, biased estimations between health outcomes and HRVs are possible. Respondents may have either exaggerated violations they experienced with hopes of increasing aid to their communities or may have censored their responses out of fear. A recall period of one year was chosen for the human rights violations module based on the likelihood of accurate reporting of traumatic events and to facilitate comparison to previous surveys in the region that also used a twelve-month recall period. [4, 5, 6, 13, 32].

Samples were selected in order to represent CBO service provision areas and do not represent states of Eastern Myanmar in their entirety as a result. Several clusters were replaced or were inaccessible as a result of security issues. Shan and Katchin States, which have been subject to conflict over the past several years, were excluded from this study. It is likely that the health and human rights situation in these inaccessible regions is worse than in those where data was collected. Thus, the estimated prevalence of human rights violations, poor health outcomes, and mortality are likely to be lower than what would have been seen had these insecure and inaccessible regions been included. Malaria and diarrhea morbidity must be interpreted in the context of the season, and thus may not be comparable to other surveys in the region conducted at different times of the year.

Mortality and morbidity estimates are an average across diverse populations in eastern Myanmar, and thus may not reflect true burden of illness within specific regions. Due to sampling limitations resulting from logistic constraints, several estimates lack precision. When comparing these estimates to regional and national estimates, this lack of precision must be taken into account.

Ideally MUAC should be complemented by weight-for-height (Z scores, WHZ) when assessing acute malnutrition in populations [36], but logistical constraints precluded anthropometric measurements necessary for WHZ, and our estimates rely exclusively on MUAC. We used both strict cutoffs as well as WHO MUAC-for-age tables to classify nutritional status, facilitating comparisons to surveys using either of these approaches.

With regards to reproductive health outcomes, due to programmatic needs of implementing partners, women between the ages of 15 and 49 who were pregnant at the time of the survey or had children under the age of 5 were surveyed in the reproductive health module. Thus, estimates of unmet need and access to reproductive health care exclude women who have never had children, women whose children have died, or women who have older children and have not had children within the past 5 years. This exclusion affects all estimates in the reproductive health module.

## Conclusion

This large survey of health and human rights demonstrates that two years after political transition, vulnerable populations of eastern Myanmar were less likely to experience human rights violations, but access to health services was still severely constrained, and risk of disease and death was still substantially higher than the country as a whole. Progress toward the elimination of human rights violations deserves recognition; however, a truly successful democratic transition requires a policy of zero tolerance and a mechanism of accountability for perpetrators. Ethnic CBOs are striving to fill the void in clinical and population health services, but dramatic increases in government and international support are necessary to ameliorate the chronic health crisis. The persistently elevated risk of child malnutrition and death continues to provide a forceful moral imperative to prioritize support for populations that remain largely outside the scope of formal government and donor programs.
